# Comparative Assessment of the Prognostic Value of Biomarkers in Traumatic Brain Injury Reveals an Independent Role for Serum Levels of Neurofilament Light

**DOI:** 10.1371/journal.pone.0132177

**Published:** 2015-07-02

**Authors:** Faiez Al Nimer, Eric Thelin, Harriet Nyström, Ann M. Dring, Anders Svenningsson, Fredrik Piehl, David W. Nelson, Bo-Michael Bellander

**Affiliations:** 1 Department of Clinical Neuroscience, Neuroimmunology Unit, Karolinska Institutet, Stockholm, Sweden; 2 Department of Clinical Neuroscience, Section for Neurosurgery, Karolinska Institutet, Stockholm, Sweden; 3 Department of Clinical Neuroscience, Section of Neuroradiology, Karolinska Institutet, Stockholm, Sweden; 4 Department of Pharmacology and Clinical Neuroscience, Umeå University, Umeå, Sweden; 5 Department of Physiology and Pharmacology, Section of Anesthesiology and Intensive Care, Karolinska Institutet, Stockholm, Sweden; University of Pittsburgh, UNITED STATES

## Abstract

Traumatic brain injury (TBI) is a common cause of death and disability, worldwide. Early determination of injury severity is essential to improve care. Neurofilament light (NF-L) has been introduced as a marker of neuroaxonal injury in neuroinflammatory/-degenerative diseases. In this study we determined the predictive power of serum (s-) and cerebrospinal fluid (CSF-) NF-L levels towards outcome, and explored their potential correlation to diffuse axonal injury (DAI). A total of 182 patients suffering from TBI admitted to the neurointensive care unit at a level 1 trauma center were included. S-NF-L levels were acquired, together with S100B and neuron-specific enolase (NSE). CSF-NF-L was measured in a subcohort (n = 84) with ventriculostomies. Clinical and neuro-radiological parameters, including computerized tomography (CT) and magnetic resonance imaging, were included in the analyses. Outcome was assessed 6 to 12 months after injury using the Glasgow Outcome Score (1-5). In univariate proportional odds analyses mean s-NF-L, -S100B and -NSE levels presented a pseudo-R^2^ Nagelkerke of 0.062, 0.214 and 0.074 in correlation to outcome, respectively. In a multivariate analysis, in addition to a model including core parameters (pseudo-R^2^ 0.33 towards outcome; Age, Glasgow Coma Scale, pupil response, Stockholm CT score, abbreviated injury severity score, S100B), S-NF-L yielded an extra 0.023 pseudo-R^2^ and a significantly better model (p = 0.006) No correlation between DAI or CT assessed-intracranial damage and NF-L was found. Our study thus demonstrates that S-NF-L correlates to TBI outcome, even if used in models with S100B, indicating an independent contribution to the prediction, perhaps by reflecting different pathophysiological processes, not possible to monitor using conventional neuroradiology. Although we did not find a predictive value of NF-L for DAI, this cannot be completely excluded. We suggest further studies, with volume quantification of axonal injury, and a prolonged sampling time, in order to better determine the connection between NF-L and DAI.

## Introduction

Traumatic brain injury (TBI) is the leading cause of death and lifelong disability in young adults worldwide, and is increasing in the elderly population [1, 2]. Despite recent advances in intensive care, morbidity and mortality remains high in severe TBI [3]. Early determination of injury severity and outcome prediction are important in order to improve care, balance benefits and risks of early treatment options, and establish predictive models that can also be used in future multi-center clinical trials. Existing predictors of outcome include age, Glasgow Coma Scale (GCS), pupil response and grading of extent/type of TBI damage on imaging [2, 4, 5]. These factors have been included in predictive models with some success in estimating outcome, although with limitations [2, 6].

In recent years, TBI research has also been devoted to finding biomarkers that can improve the predictive capacity of these demographic, clinical and imaging factors, but also to highlight relevant disease pathways (recently reviewed in [7, 8]). The most extensively studied biomarker in TBI is S100B, with numerous studies showing a strong predictive capacity, also in the multivariate setting, and correlation to intracranial pathology [9, 10]. However, S100B is expressed in other cell types such as melanocytes, Langerhans cells, Schwann cells, chondrocytes and adipocytes and its serum concentration can increase due to extracranial injuries, such as bone fractures (including skull and facial bones) and muscle injuries [11–15], as well as in multitrauma patients without head injury [16]. Also, S100B is found mainly in astrocytes and may not reflect the extent of damage in neurons, which is more prominent in certain injury types, such as diffuse axonal injury (DAI) [17]. NSE is an isoenzyme of enolase, a glycolytic protein that is present, predominantly, in the cytoplasm of neurons, neuroendocrine cells [18] and erythrocytes [7, 19]. As NSE is present in erythrocytes, its usefulness as a serum biomarker for brain damage is limited since hemolysis will provide elevated levels of non-cerebral origin [7, 20]. A more clinically useful marker for axonal injury is thus warranted.

Neurofilaments consist of three kinds of chains, neurofilament light chain (NF-L) of 68 kDa, a neurofilament intermediate chain of 150 kDa, and a neurofilament heavy chain (NFH) of 190 to 210 kDa, and assemble to form intermediate filaments of 10μm diameter, which are the main components of the axonal cytoskeleton. In situations of axonal damage they can be released and measured in biological fluids [21]. NF-L has emerged as a promising cerebrospinal fluid (CSF) biomarker for neurological disability and neuroaxonal damage in neurodegenerative and neuroinflammatory diseases, such as amyotrophic lateral sclerosis and multiple sclerosis, respectively [12, 22–24]. It is also increased in CSF following concussions related to boxing, in the extracellular space of pericontusional areas in TBI and in the serum of patients with spinal cord injury (SCI), where it correlates with outcome [25–27]. In aggregate, NF-L shows promise as a serum biomarker for TBI, with a different cellular origin than S100B.

By using a panel of several biomarkers of brain injury, the predictive capabilities toward long term outcome has been shown to increase [28]. In a study from Mondello et al, it was also shown that a biomarker of neuronal origin (ubiquitin carboxy-terminal hydrolase L1, UCH-L1), combined with a marker of glial origin (glial fibrillary acidic protein, GFAP) could discriminate between diffuse and focal injury on admission, and the ratio between the two proteins was a better outcome predictor than either protein alone [29]. Presumably, monitoring several biomarkers, that reflect different clinical information, would improve the management of patients suffering from TBI.

The main objective of this study was to analyze a possible predictive value of CSF and/or serum NF-L, as compared and adjusted to other known predictive factors including the two biomarkers S100B and NSE. Secondary objectives were to explore the levels of NF-L after TBI in serum and CSF and to investigate if NF-L and S100B reflect different pathophysiologic phenomena as analyzed by neuroradiology. Additionally, in an exploratory approach, we also looked for factors that might explain the increase of NF-L after TBI. Our study revealed a prognostic value for serum levels of neurofilament light, independent from S100B, towards long term functional outcome following TBI.

## Methods

### Clinical cohort

Patients with NF-L samples in the Traumatic Brain Injury Database at Karolinska University Hospital, between 2007 and 2013, were retrospectively enrolled. Time points were chosen to correspond to another unpublished study analyzing genetic differences in TBI patients (#2005/1526/31/2). The patient cohort partially overlaps with an earlier study investigating the prognostic relevance of S100B levels [10].

### Ethic statement

The patients were included retrospectively (2007–2013), thus no verbal or written consent could be acquired. Clinical data is stored in the patient’s hospital charts, which are biometrically protected and stored on hospital servers. The extracted data was analyzed anonymized, and all the results were presented on a group level, hence making it impossible to identify individual patients. The study was approved by the Ethical Vetting Board of Research Involving Humans, Stockholm, Sweden (approval number 2014/2025-31). Both the current ethical application and the one approved for the other study (#2005/1526/31/2) approve that S100B, NSE and NF-L are sampled in serum and CSF.

### Admission parameters

Glasgow Coma Scale (GCS) [30] was assessed at admission to the hospital. Pupil responsiveness was used as a factor; 0 = pupil responsiveness, 1 = unilateral unresponsiveness and 2 = bilateral unresponsiveness. Injury Severity Score and Abbreviated Injury Score were assessed following admission to the emergency room [31, 32].

### NF-L

Determination of serum and CSF levels of NF-L were carried out using a commercially available ELISA kit (Uman Diagnostics, Umeå, Sweden) according to the manufacturer’s instructions. Measurements were performed in duplicates using 50 μl undiluted cell-free serum, or 10μl CSF per well. The detection cut-off is set to 31ng/L, the standard is ranging from 100 to 10,000 ng/L. When values were below or above the ELISA detection range, the sample was re-run at a lower or higher dilution, respectively, thus the concentration was never “0” (zero). NF-L has been shown to be a stable analyte and the measurements are not affected by repetitive thawing (up to 4 thaws) [33]. Five serum and 7 CSF samples were thawed a second time to run repeat readings. Other than that, all samples were thawed only once.

### S100B and NSE

All serum S100B samples collected until September 2008 were analyzed at Karolinska University Hospital, Department of Clinical Chemistry, using a quantitative automated luminometric immunoassay (LIAISON-mat S100 system, Diasorin, Sangtec, Italy). In September 2008, the Department changed to an automatic electrochemiluminescence immunoassay (Elecsys S100B; Roche Diagnostics, Penzberg, Germany) analyzing method. A good correspondence between the two methods has been shown, including internal validation by the Department of Clinical Chemistry, Karolinska University Hospital, Solna, Sweden [34, 35]. Serum NSE, as well as CSF levels of S100B and NSE, were analyzed using the immunoradiometric assay (Liaison, DiaSorin, Italy) throughout the whole study at the Karolinska University Hospital, Department of Clinical Chemistry. The detection levels for the Liaison ranges from 0.04 μg/L for NSE and 0.02 μg/L for S100B, while the Elecsys detects serum S100B levels down to 0.005μg/L. No patient presented with concentrations below detection levels. The serum levels and clinical data were collected from the digital medical files in the hospital database system Take Care (CompuGroup Medical Sweden AB, Farsta, Sweden) for each patient. As S100B and NSE are routinely sampled twice daily in our clinic, a mean level of sample measurements acquired during the day of NF-L-sampling was used. For serum S100B, the peak level 12–36 hours after TBI and for serum NSE, the peak level from the first 48 hours after trauma was also acquired [36]. CSF levels of S100B and NSE were noted, if available the same day as the CSF NF-L samples.

### Neuroradiology

Computerized tomography (CT) scans of each patient were used to compute the Stockholm CT score [37], the Marshall classification [38] and the Rotterdam CT score at admission (initial CT scan) [39]. The Marshall classification primarily describes if the injury is diffuse, with different degrees of severity, or focal. The Rotterdam system is graded in different levels of severity (higher level = increased risk of unfavorable outcome) and includes compression of basal cisternae, >5 mm of midline shift and the presence of subarachnoid hemorrhage. Stockholm CT score uses midline shift as a continuous variable, but also looks at the presence of diffuse axonal injury visible on CT scans and subarachnoid hemorrhage. Both Rotterdam and Stockholm CT-scores considers the presence of epidural hematoma to be a favorable outcome factor. The analysis protocol also included volumes and locations of contusions, infarctions and information on presence and extent of DAI lesions. Additionally, 85 patients had undergone magnetic resonance imaging, (MRI) which was analyzed using the same protocol. The clinical MRI protocol included Echo Planar diffusion-, Fluid Attenuated Inversion Recovery- (FLAIR), Gradient Echo- (GRE), and T1- and T2 weighted image sequences. MRI was performed in individual patients according to the clinical course and was usually used to assess if diffuse axonal injury was present in the more severe cases of TBI. MRIs were graded according to Adams et al [40], determining grade and type of DAI, where grade 1 = subcortical DAI, grade 2 = basal ganglia and/or corpus callosum (as well as subcortical), grade 3 = brain stem involvement (as well as grade 1–2 locations).

### Outcome

Outcome was evaluated by Glasgow Outcome Score (GOS) [41] 6–12 months post trauma. This is regularly performed at our department, either at a follow-up visit at the neurosurgical department or rehabilitation clinic approximately 6 months after trauma and/or by a structured questionnaire 12 months after trauma, therefore a combination of assessed and self-reported outcome was used. The latest available GOS score was used if assessed at more than one time point. GOS 1 indicates death, GOS 2 vegetative state, GOS 3 severe, dependent state, GOS 4 moderate, independent state, and GOS 5 full recovery. In outcome prediction models, all steps of GOS was used, thus no dichotomization was performed.

### Statistical analyses

The statistical progam R (v3.1.0, R-Foundation for Statistical Computing, Vienna, Austria; http://www.R-project.org) was used with the “lrm” package for proportional odds models. Parameters known to be predictive of TBI outcome were included as adjusting parameters. These included Age, GCS, Pupil response, CT score, and trauma grade (ISS, AISS), which subsequently are referred to as core variables [37]. Since data on NF-L was sampled 1–3 times per subject and at different time points, mixed model analyses were not feasible. Instead median, mean and max values were calculated and used in the statistical models. Logarithm values for NF-L, S100B and NSE data were used to near a normal distribution of these variables. Multiple Imputation (MI) was used to impute missing data, imputing 7 complete sets, and project the composite introduced uncertainty of imputation in the multivariate models [42]. Multivariate proportional odds analyses were performed towards GOS. Outcomes for different levels of NF-L and S100B were illustrated using conditional density plots. Accuracy of models were evaluated using Nagelkerke’s pseudo-R^2^ [43], bootstrapped for bias when the number of predictor variables are increased. Nagelkerke’s pseudo-R^2^ provides an estimated explained variance, a number between 0 and 1 where 1 is a fully explained model. The explained variance of linear models identifying factors related to serum (s)-NF-L and CSF-NF-L is given as an adjusted R^2^. A Mann-Whitney U-Test was used to compare NF-L levels between patients with and without DAI in Marshall Grade II patients. The raw data used in this study is available as supplementary information (S1 File).

## Results

### Demographics

S-NF-L samples were collected in 182 patients (75% males). The median initial GCS was 6. Demographic histograms of primary core data and outcome are shown in Table 1. The early GCS distribution showed a bias towards lower scores, in part explained by many patients being sedated and intubated directly at the scene of accident. There were no patients with GOS2 (vegetative state) in the data set. For the 159 patients that survived, outcome was assessed at 6–7 months (n = 28), 8–10 months (n = 18), but primarily at 12 months (n = 116, 73%) after trauma. Uninjured reference controls are presented in Table 1 for serum and CSF S100B, NSE and NF-L levels. The data set was largely complete for core parameters, s-NSE and s-S100B, and outcome (Table 2).

**Table 1:** Patient demographics.

Age (median- 1st/3rd quartile)	55 (37–63)
Glasgow Coma Scale (mean—SD)	7 (4)
GCS 3–8	n = 128 (71%)
GCS 9–13	n = 39 (21%)
GCS 14–15	n = 15 (8%)
Abbreviated Injury Severity Score (AIS) (mean—SD)	4 (1)
Injury Severity Score (ISS) (median- 1st/3rd quartile)	25 (19–29)
Pupil responsiveness	
Bilateral responsive	n = 136 (75%)
Unilateral unresponsiveness	n = 37 (20%)
Bilateral unresponsiveness	n = 7 (4%)
Missing	n = 2 (1%)
Neuroradiology	
Initial computerized tomography (CT) scan	
Marshall Score	
Grade I	n = 0
Grade II	n = 67 (37%)
Grade III	n = 11 (6%)
Grade IV	n = 2 (1%)
Grade VI	n = 100 (55%)
Diffuse injury (Grade I-IV)	n = 80 (44%)
Missing	n = 2 (1%)
Rotterdam Score	
Grade 1	n = 4 (2%)
Grade 2	n = 21 (12%)
Grade 3	n = 79 (43%)
Grade 4	n = 32 (18%)
Grade 5	n = 33 (18%)
Grade 6	n = 13 (7%)
Missing	n = 2 (1%)
Stockholm Score (mean—SD)	2.53 (1.06)
Magnetic resonance imaging (MRI)	n = 85 (47%)
Diffuse Axonal Injury (DAI)	n = 40 (47%—of performed MRI)
DAI grade 1	n = 6 (15%—of all DAI)
DAI grade 2	n = 14 (35%—of all DAI)
DAI grade 3	n = 20 (50%—of all DAI)
Serum levels	
S100B, 12-36h after trauma (median- 1st/3rd quartile) (μg/L)	0.46 (0.22–0.81) (Ctrl: 0.02, 0.00–0.13)*
NSE, peak level (median- 1st/3rd quartile) (μg/L)	24 (18–33) (Ctrl: 9.4, 6.3–13.3)*
NF-L (median- 1st/3rd quartile) (ng/L)	400 (181–865) (Ctrl: 7.9, 5.6–17.2)*
Cerebrospinal fluid levels	
S100B, n = 68, pooled samples n = 117 (median- 1st/3rd quartile) (μg/L)	15.5 (2.6–63.8) (Ctrl: 0.96 ± 0.50)‡
NSE, n = 67 patients, pooled samples = 117 (median- 1st/3rd quartile) (μg/L)	35 (14–108) (Ctrl: 6.43 ± 4.10)‡
NF-L, n = 84 patients, pooled samples = 167 (median- 1st/3rd quartile) (ng/L)	7026 (2610–19204) (Ctrl: 138 ± 31)‡
Outcome	
NICU Length of stay (median- 1st/3rd quartile) (days)	12 (6–21)
Glasgow Outcome Score (GOS 1–5)	
GOS 1 –Dead	n = 23 (13%)
GOS 2—Vegetative state	n = 0
GOS 3—Severe disability	n = 68 (37%)
GOS 4—Moderate disability	n = 54 (30%)
GOS 5—Mild disability	n = 37 (20%)

Illustrating patient demographics including CT, MRI, biomarker and outcome data. Reference concentrations from healthy controls (Ctrl) are presented for each biomarker in serum and CSF [44–47].

* = median, IQR

‡ = mean, SD

**Table 2:** Missing data.

Core	Percent missing	Biomarkers	Percent missing
Age	0	Serum (s)—NF-L	0
Pupils	1.1	s-NSE peak	0
GCS	0	s-S100B 12h-36 hours	0
CT	1.1	s-NSE (day of NF-L sample)	3.3
AISS/ISS	1.1	s-S100B (day of NF-L sample)	3.3
GOS	0		

The amount of missing data was low and was imputed using multiple imputations.

### Characteristics of s-NF-L

**Fig 1:**
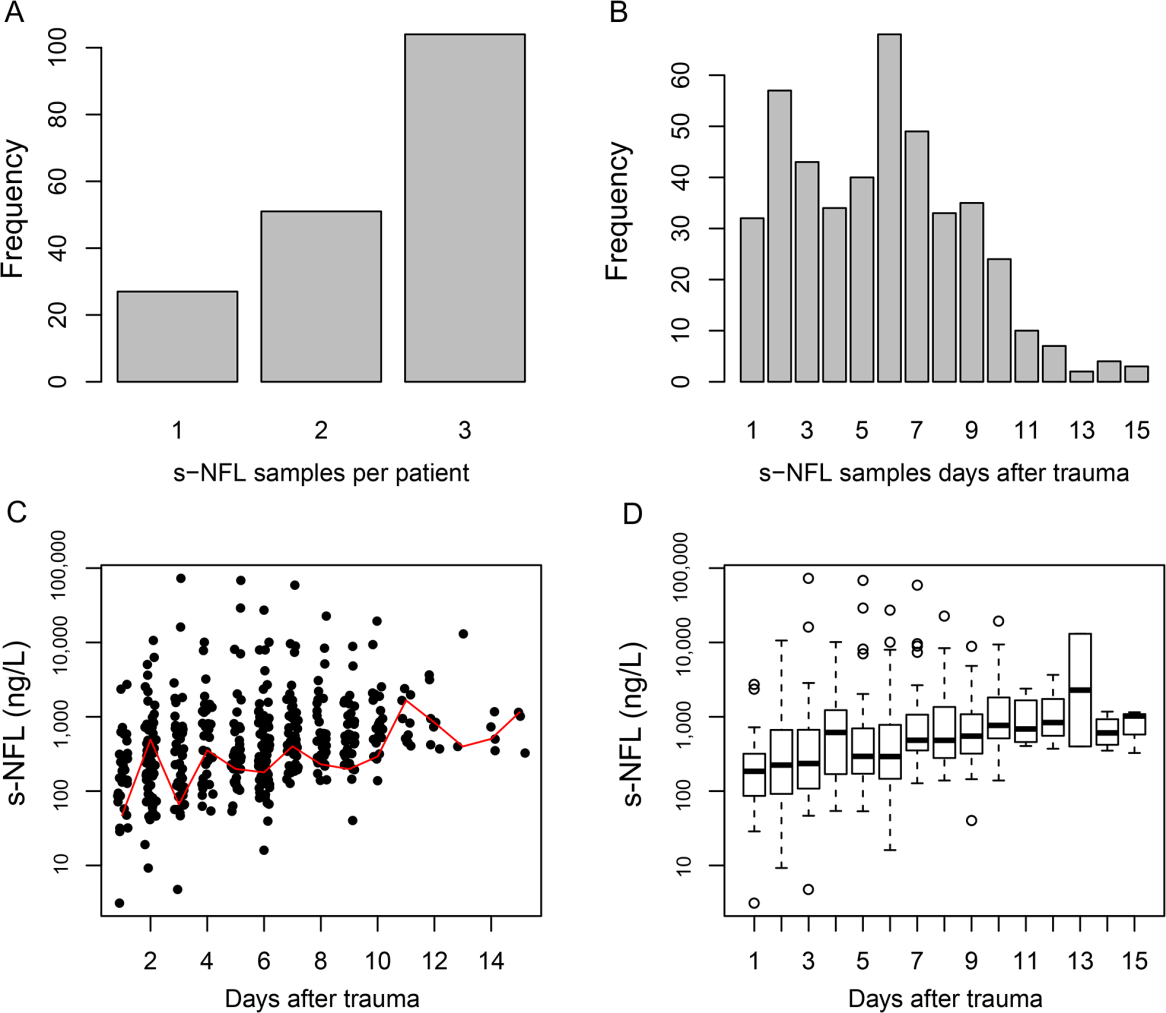
Characteristics of serum NF-L samples. Histograms illustrating the number of s-NF-L samples per patient (A) and the distribution over time after trauma (B). C illustrates the s-NF-L levels over time (one dot per sample), with a red line representing the locally weighted scatterplot smoothing (LOWESS), a nonlinear regression of data points. D illustrates the s-NF-L levels over time after trauma using boxplots.

**Fig 2:**
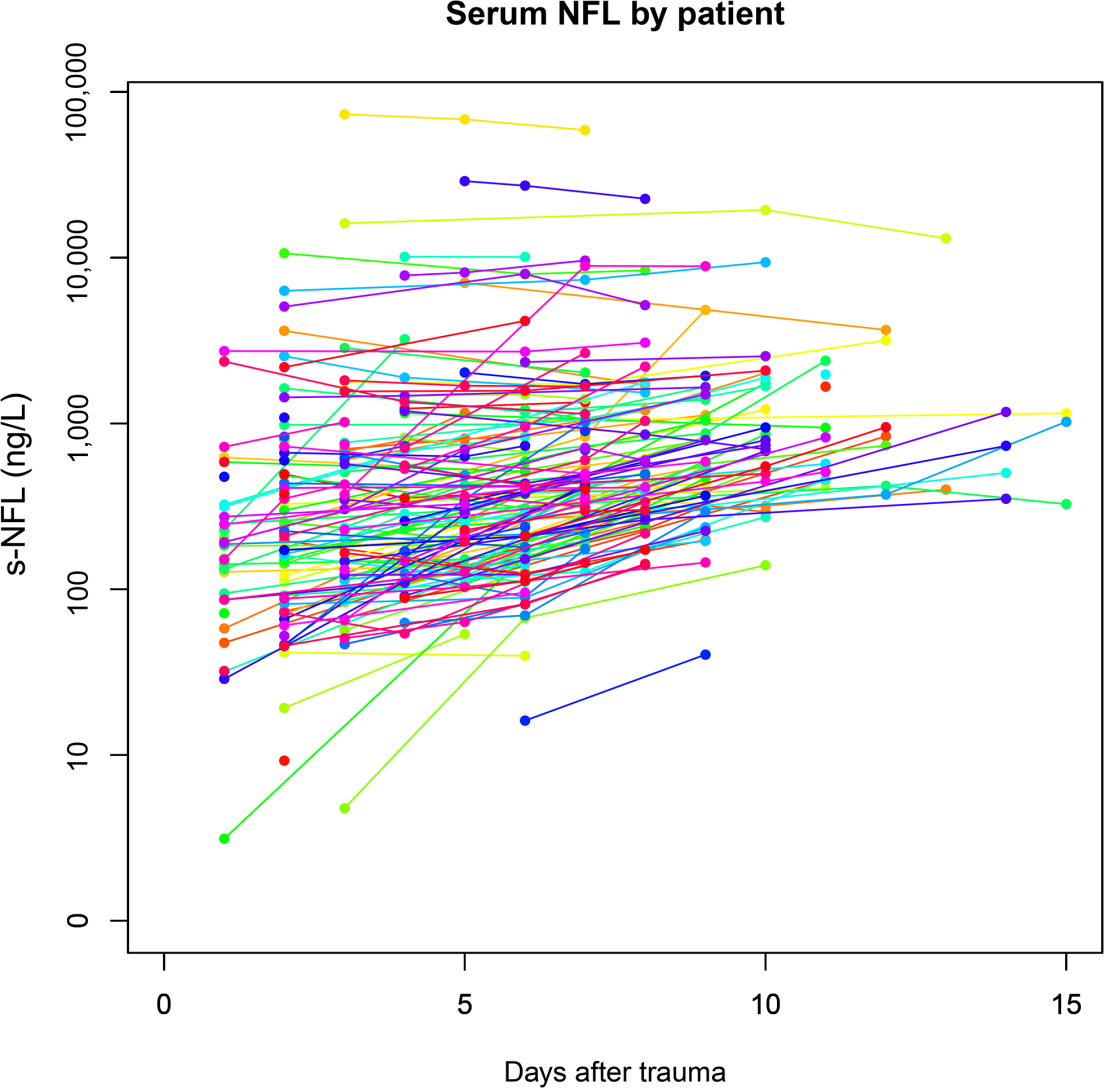
Serum NF-L levels by patient. Every line (separate color) represents an individual patient. Generally, even if there were differences in concentration between patients they were limited over time for the individual patient.

Between 1 and 3 samples were collected per patient, as available (Fig 1A and 1B). In total, 439 serum-, and 167 CSF samples, of NF-L were acquired. There was a general upward trend of s-NF-L at the group level over time (Fig 1C and 1D), but differences on an individual basis were often less pronounced (Fig 2). Since mixed models were not applicable, and as intra-individual changes were limited, data was reduced to mean and max per patient. Additionally two variables were derived: a variability parameter of the standard deviation per patient and a time parameter stratifying samples to early (1–5) and late (5–15) sample days, respectively. The analyses of these two parameters are affected by missing data points and should be considered as exploratory.

### Univariate analyses–outcome prediction

Univariate proportional odds were determined for core and biomarker predictor variables in relation to GOS (Table 3). Mean, median and max values were determined for s-NF-L, s-S100B, s-NSE, as well as the s-S100B/s-NF-L ratios (mean per patient), for each patient. S100B was seen to be the best univariate predictor of outcome. The Stockholm CT score was more informative than Rotterdam or Marshall, and therefore chosen as the core CT variable in the multivariate analyses. The AISS, similarly, was chosen over the ISS. Gender was not significant. All other variables were significant (p<0.05) except for the Marshall classification and, surprisingly, the GCS. The derived NF-L variability and the early/late NF-L parameter were found to be non-significant.

**Table 3:** Univariate proportional odds analysis of parameters versus Glasgow Outcome Score.

Core variables	Pseudo R^2^	Biomarker variables	Pseudo R^2^
Age	0.167	S—NF-L mean/max	0.062/ 0.062
GCS	0.016 (p = 0.10)	S—S100B mean/max	0.214/ 0.193
Pupil response	0.033	S—NSE mean/max	0.074 / 0.062
Stockholm score	0.119	S—S100B 12 h	0.179
Rotterdam score	0.05	S—NSE peak	0.045
Marshall class	0.021 (p = 0.06)	S—NSE at S100B (12-36h)	0.053
AISS/ISS	0.067 / 0.025	S—S100B/s-NF-L ratio	0.204

Individual parameters and correlation (pseudo-R^2^) toward long term functional outcome (GOS).

**Fig 3:**
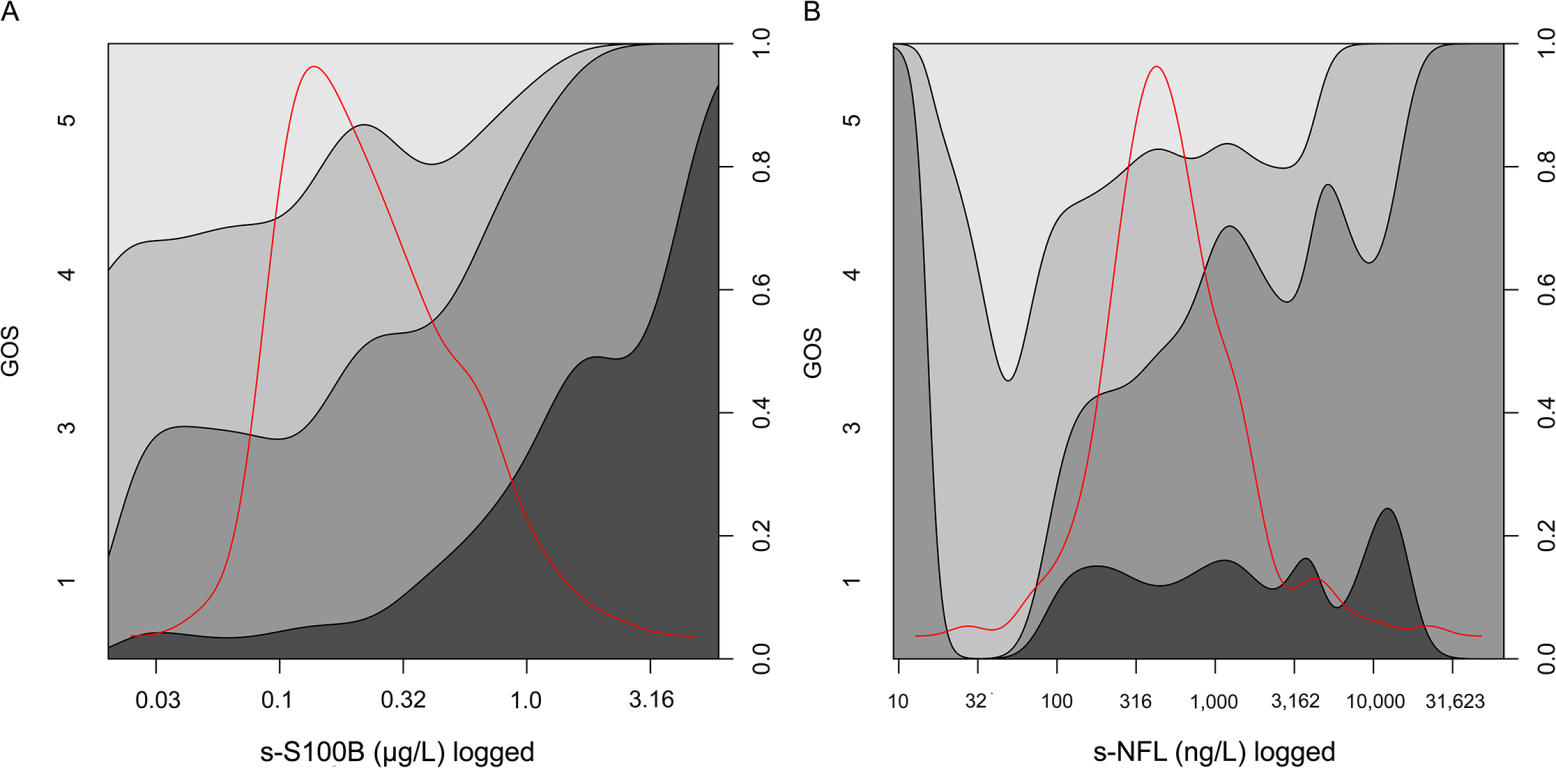
Both serum-S100B and –NF-L correlate to TBI outcome. Serum levels of S100B (A) and NF-L (B) (x-axis, respectively) vs Glasgow Outcome Score (GOS) (y-axis, left) shown using conditional density plots. The red line represents the data distribution. Outcome proportions are illustrated, summing to one (y-axis right).

GOS spread at all levels of s-S100B (mean per patient) and s-NF-L (mean per patient) are shown in Fig 3, also including a data density distribution in red with no relation to the y axis. As evident from the graph, the relation of S100B levels to GOS is more consistent for all outcome levels than s-NF-L, which however differentiates better outcome levels.

### Multivariate analysis–outcome prediction

For multivariate analyses, core data and mean serum biomarker data were imputed with MI with ≤3.3% missing data for any variable. Missing values were non-significant towards GOS, supporting the missing-at-random assumption of imputation. A correlation matrix indicated that s-S100B and s-NSE were most highly correlated (R^2^ = 0.26, p<0.00001).

The core parameters (Age, GCS, Pupil response, Stockholm CT score, and AISS), similar to parameters presented by the IMPACT group [43], exhibited an adjusted pseudo R^2^ of 0.25 in a proportional odds models towards GOS. The individual biomarkers s-S100B, s-NSE and s-NF-L added to this model increasing the estimated explained variance of models to 0.33, 0.27 and 0.30, respectively. A step-up procedure found both s-S100B and s-NF-L significant in a combined model exhibiting an adjusted Pseudo R^2^ of 0.35 and thus adding a partial R^2^ of 0.098 to the base model, where s-NF-L adds an additional 0.023 over S100B. The difference in deviances of this model and sub-model were tested and support a significantly better model when combined (p = 0.006). These results indicate that both s-S100B and s-NF-L are significant and independent predictors of TBI outcome.

### Relations of s-NF-L and CSF-NF-L

**Fig 4:**
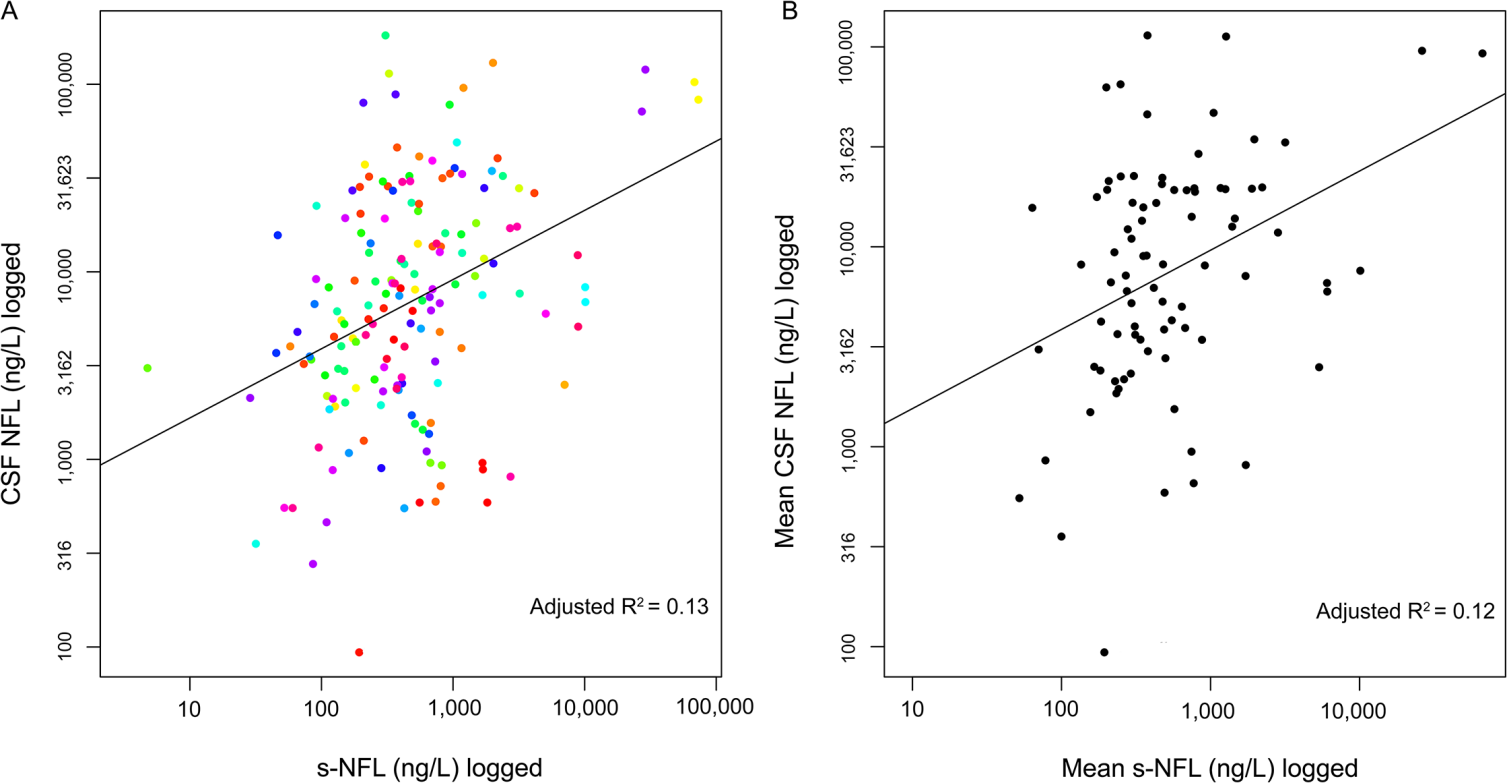
Correlations between CSF and serum samples of NF-L are illustrated. One color represents one patient (A). The mean CSF and serum samples were calculated and correlated (B).

In a subset of 84 patients who had ventriculostomies for intracranial pressure monitoring, NF-L was also measured in CSF. The relatively weak but significant relations between serum and CSF-NF-L are seen in Fig 4, exhibiting an adjusted R^2^ of 0.12 (p<0.001). In comparison, the logged serum and CSF correlation (R^2^) for S100B and NSE were 0.34 and 0.17, respectively.

**Fig 5:**
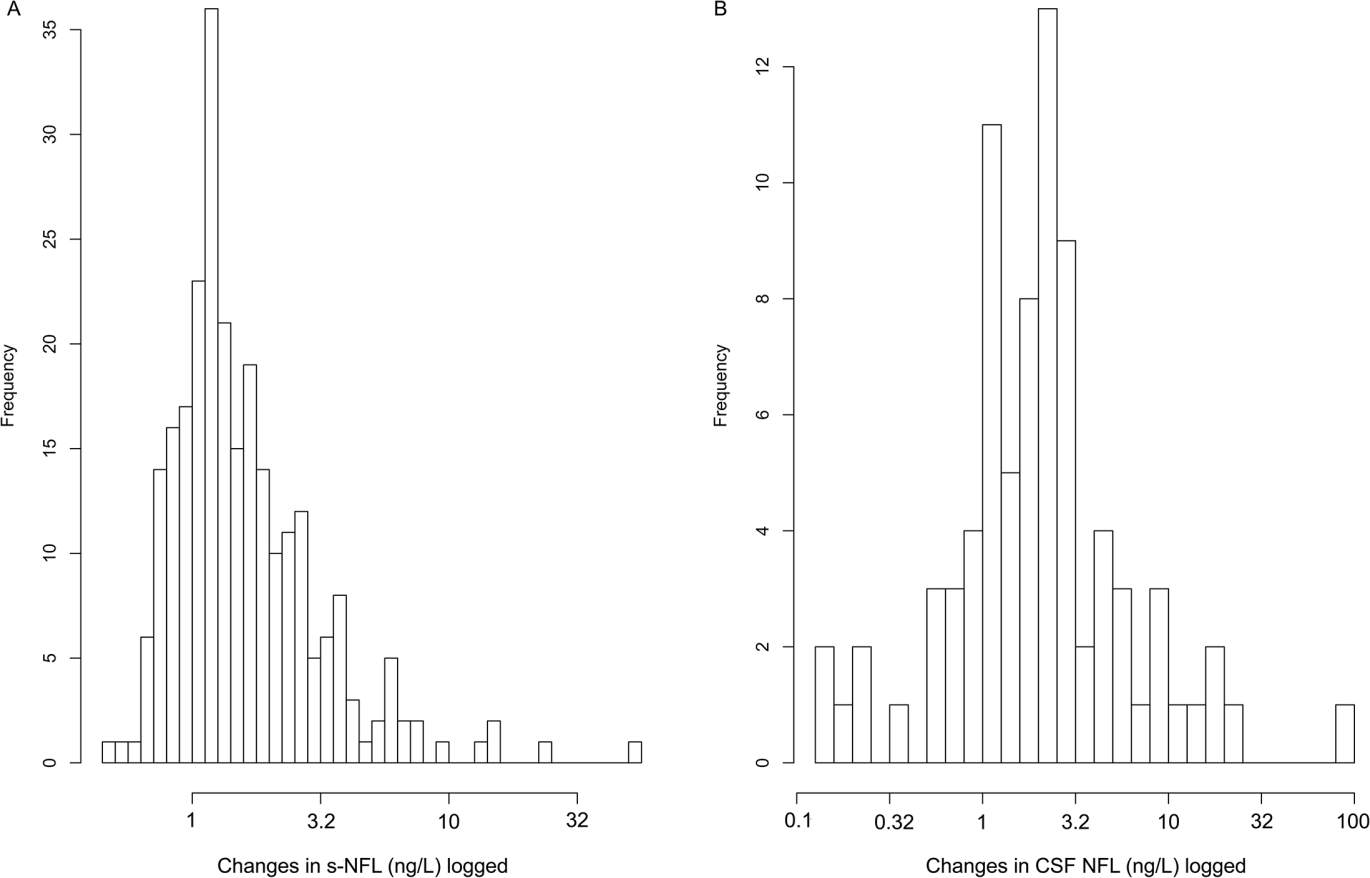
The within patient changes of serum (A) and CSF (B)-NF-L illustrated using histograms of logged data. The differences over time for each patient were low, with a majority of patients not diverging more than 3.2 ng/L in serum (Fig 5A) and more than 10 ng/L in CSF (Fig 5B).

When analyzing the ratios of CSF vs. serum values of NF-L it was found that the between individual ratios differ greatly, whereas the intra-individual changes are generally more moderate and with no clear time dependency (data not shown). Levels of both s-NF-L and CSF-NF-L were highly related to the day of sampling (p <0.0001) with a positive correlation coefficient and CSF-NF-L levels, as s-NF-L, generally increased as seen in a histogram of changes in Fig 5.

### Analyses of CSF-NF-L cohort

In the subset (n = 84) of patients with CSF-NF-L similar univariate and multivariate analyses were performed. Caution must be made when comparing with the results in Table 3 with the analysis in this smaller subgroup of patients, which comprised a more severely injured cohort requiring intra-cranial pressure monitoring. The CSF-NF-L was found to be significantly related to GOS in a univariate proportional odds analysis (p = 0.0056, pseudo-R^2^ 0.098), however non-significant when adjusted for the other variables of the base model, with or without S100B.

### Relations of s-NF-L to predictors and imaging

Parameters that significantly and positively correlated to s-NF-L (logged, mean per patient) in univariate analysis withstanding a Bonferroni correction for multiple testing were: Days-after-trauma when the serum sample was collected (p = 0.00037), s-NSE (peak first 48 hours, logged, p = 0.00080) and GOS (p = 0.00036). Additional parameters significant in univariate analyses (without Bonferroni correction) and included in an initial multivariate model prior to step-down were, ISS (p = 0.017) and s-S100B (logged, p = 0.0010) at 12 hours post trauma. No CT scores or individual components thereof were significantly correlated to s-NF-L, while serum levels of S100B were (Stockholm CT score, p = 0.0038). Only Days-after-trauma and GOS remained significantly related to s-NF-L in multivariate analyses.

When analyzing the smaller cohort of patients that underwent MRIs (n = 85) midline-shift on MR was significantly related to s-NF-L (p = 0.012). However, midline-shift was not significantly correlated to s-NF-L in early CTs in the full cohort, or a subgroup of patients with late CT’s (n = 102). The MRI population is also noted to have a lower mean age and GCS. Notably, s-NF-L was not related to outcome in this smaller MRI cohort. Specifically, no significant relations between DAI locations, or grade, on MRI vs. s-NF-L, were identified.

In an exploratory attempt to remove focal injuries, a subgroup analysis, including only Marshall Grade 2 (diffuse injury, n = 40), including diffuse TBI patients with DAI (n = 29) and without (n = 11) on MRI, there was no significant difference between the presence of DAI and NF-L median levels in serum (p = 0.2664) and CSF (p = 0.5564), respectively (Mann-Whitney U Test).

### Relations of CSF-NF-L to predictors and imaging

In the subpopulation of patients with sampled CSF-NF-L (n = 84), multivariate analyses identified age, days-after-trauma, s-NF-L and CSF-S100B or CSF-NSE (interchangeably, but not simultaneously in model due to co-variation) to be independently correlated to CSF-NF-L, with the model exhibiting an adjusted R^2^ of 0.35. GOS was not found related to CSF-NF-L in this cohort. In the group of patients with both MRI and CSF-NF-L (n = 47), no MRI variable was correlated significantly to CSF-NF-L levels.

## Discussion

We determined serum and CSF levels of NF-L in a large cohort of retrospectively collected samples from TBI patients, and correlated the levels to functional outcome while adjusting for established predictive factors. This study represents the first larger study of this biomarker in the context of human TBI, where previously only a small study revealed a correlation between microdialysis-based measurements of the brain extracellular fluid levels of NF-L and outcome [25]. We detected a significant correlation to outcome, which was independent, though with inferior predictive value to S100B. Both serum and CSF levels of NF-L were stable, but with an upwards trend during the first 15 days after trauma. The correlation between serum and CSF NF-L levels was weak, yet significant. However, we did not observe any correlation to MRI imaging parameters and thus no clear relation to a specific type and/or grade of diffuse injury.

To the best of our knowledge, this is the first study to sample NF-L in human serum following TBI. NF-L, in contrast to S100B, is a neuronal protein, and thus would perhaps better reflect loss of nerve cells and axonal connections, an important determinant of long-term deficits in TBI [48]. We therefore, determined the predictive value of NF-L both in univariate and multivariate analyses, where S100B was included, and found that both S100B and s-NF-L significantly contribute to outcome prediction, albeit s-NF-L levels contributed less than serum levels of S100B. This additive predictive value of s-NF-L indicates that NF-L could be used as a biomarker for predictive models of TBI outcome. The additional partial R^2^ of NF-L in the prediction model is 0.023, which is lower than age, pupil responsiveness and GCS motor score in outcome models using the IMPACT study material [43] and what S100B provides in outcome prediction models by our group [10]. However, even if it is only a few percentage points, that two biomarkers used together provides independent information in outcome prediction models is a major finding. In previous studies, combinations of biomarkers have been shown to increase outcome prediction (GFAP+S100B, NSE+S100B+GFAP and UCHL-1+GFAP) and this is accordingly suggested for NF-L+S100B in our study [28, 29, 49]. A predictive value of NF-L for TBI outcome is also supported by previous data which showed that extracellular fluid levels of NF-L in severe TBI patients correlate to TBI outcome [25] and that s-NF-L levels also predict SCI outcome [27]. On the other hand, we only found a significant prediction between CSF-NF-L and outcome in a univariate analysis, presumably because the material was underpowered for such an analysis. CSF-NF-L levels were correlated to CSF-NSE and CSF-S100B levels, indicating a correlation to injury severity. Studies detecting a correlation between injury severity in mild TBI and CSF-NF-L used earlier/later sampling, making comparisons to our results difficult [26, 50]. In aggregate, an independent predictive capability of s-NF-L towards outcome was shown, even when adjusting for S100B values, perhaps indicating that a separate pathological process, including axonal injury, was being monitored.

Although we found a correlation of NF-L levels to ISS, which is partly influenced by intracranial injury, we could not verify an association of NF-L to the extent of DAI on MRI or to damage assessed using CT scores. This could be due to analyses difficulties because of the heterogeneity of disease and sample size in our study. Another reason could be how NF-L behaves physiologically in focal vs diffuse injuries. In focal lesions, NF-L might be rapidly released to the CSF, while in diffuse injuries, it may first accumulate during axonal swelling, intermittently disrupted axons and be subsequently released over longer time periods [51]. However, an attempt to only look at diffuse injuries on CT scan and DAI on MRI, removing patients with focal injuries, failed to yield any statistical correlation to NF-L levels. Another reason why DAI did not yield significantly higher levels of NF-L could be the way that DAI is quantified. Even if we noted localizations affected by DAI on MRI, the exact volume was not available. NFH has been seen to increase in serum relatively more in diffuse injury compared to focal injuries [52, 53], unlike NF-L in our study. While midline shift on MRI was correlated to s-NF-L, we could not see any correlation to CT data when including all patients, suggesting that this positive correlation was probably a result of multiple testing in different subpopulations. S100B was correlated to the extent of CT injury, whereas NF-L was not, indicating that conventional neuroradiology cannot adequately monitor the pathophysiological process leading to increased NF-L levels. Also, it is difficult to determine the correct extent of axonal injury using CT and MRI, so perhaps novel techniques, such as high resolution fiber tracking [54], are needed to better determine the full extent of the injury.

Contrary to the MRI findings, the finding that the predictive value of NF-L is independent of S100B suggests that NF-L might be used in models to monitor an additional pathophysiological pathway. NF-L, in contrast to S100B, is a neuronal protein, and thus should better reflect loss of nerve cells and axonal connections, an important determinant of long-term deficits [48]. This might be of special relevance for discriminating different injury types, e.g. diffuse injury vs focal tissue injury, as shown by Mondello et al by using the ratio GFAP:UCH-L1 [29] or disclosing special neuro-degenerative/inflammatory pathways. In an exploratory approach, as our data were not completely suited for such an analysis, we did not find a S100B:NF-L “glial:axonal” ratio to enhance predictions in outcome. One of the reasons this is difficult is probably due to the extreme difference in half-life between the two proteins (1 hour vs 3 weeks) [55, 56] compared to GFAP and UCH-L1 (16 vs 8 hours) [57, 58]. However, this cannot be excluded and should be evaluated in future studies with more controlled sampling times, presumably early after trauma. NSE was more highly correlated to levels of NF-L, compared to S100B, hence supporting NF-L as a marker of neuronal injury. However, NSE would probably be less specific than NF-L in TBI as extracranial sources exist and hemolysis in samples is a problem [11, 19]. Even if DAI was not correlated to NF-L levels in serum and CSF in our study, we believe that prospective multi-center studies are needed to determine the correlation between axonal injury and NF-L levels.

While S100B and NSE are present in extracranial tissue,[11, 19] there are no studies reporting extracranial origins of NF-L. Unfortunately, no specific extracranial injury data was available in this study to confirm this, with the ISS and AIS being too influenced by the intracranial injury. As could be seen in relation to uninjured reference controls, all CSF and serum levels of the biomarkers were elevated, which was especially true for the NF-L levels. The CSF- and s-NF-L measurements in our study (median 7026 ng/L and 400ng/l respectively) are similar to previously reported data in mild TBI and severe SCI. Regarding CSF, the levels are higher than in a previous study by Zetterberg et al analyzing NF-L in CSF of boxers suffering mild TBI (mean 845 ng/L) [50] and s-NF-L levels are similar to those reported by Kuhle et al in severe spinal cord injury (70–800 ng/L) [27]. On the other hand, the correlation between CSF-NF-L (log) and s-NF-L (log) in our study only had an adjusted R^2^ of 0.13. In contrast, serum-CSF (log) correlation (R^2^) for S100B and NSE in this study were 0.34 and 0.17 respectively, thus NF-L presented similar results as NSE. As this is the first study measuring both s- and CSF-NF-L in CNS trauma, we can only compare to a R^2^ value measured in other neurological diseases (Alzheimer’s disease, Guillain-Barre syndrome and amyotrophic lateral sclerosis) which showed a stronger correlation (R^2^ = 0.46) [24]. This discrepancy could partly be explained by the heterogeneity and severity of the TBI compared to neurodegenerative diseases, as well as, a higher variation in the permeability of the blood-brain barrier (BBB). However, since the NF-L levels of CSF and serum have a strong correlation to the parameter days sampled after injury, they probably correlate more to the extent of the injury than to the BBB integrity, which has been shown regarding S100B levels [59, 60] and are presumably excreted predominantly from the brain using the recently discovered glymphatic system [61]. Also, animal studies show that the BBB is primarily affected only the first 30 minutes following experimental TBI [62, 63], hence earlier than when samples were acquired in this study. Moreover, CSF-NF-L levels were correlated to age, perhaps indicating a higher morbidity of neurodegenerative diseases in this material including relatively old patients (median age 55 years). In aggregate, looking at other studies of NF-L, our levels seem plausible and the discrepancies could be explained by differences in injury severity and the heterogeneity of TBI.

Generally, logged NF-L levels remained unchanged, or increased, during the first days after injury. Being an obvious effect of logarithmic data, this could also be explained by the half-life of NF-L in vivo, which has been shown to be about three weeks [64], and hence considerably longer than for NSE [56] (approximately 20 hours) and S100B [55] (1 hour). Thus, if the NF-L levels are elevated initially, they will probably remain high, throughout the first two weeks after TBI. This means that NF-L levels, measured within 2 weeks, will likely be heavily influenced by the primary brain injury and the very early pathophysiological cascades. Probably due to its short half-life, S100B has been shown to be more dynamic and thus perhaps better as a biomarker to detect secondary injuries in the neuro-intensive care unit [65]. Still, the increase of NF-L in individual patients might indicate acceleration in neuronal death/axotomy due to secondary effects of inflammatory or neurodegenerative pathways, which in turn could also depend on genetic factors. This is supported by our previous data in an animal TBI model on two different inbred rat strains, where genetic heterogeneity and neuroinflammatory pathways had an effect on CSF-NF-L values [66]. Further studies would therefore be needed in order to discover the potential source of this temporal increase in NF-L.

### Limitations

The number of NF-L samples acquired, per patient, in this study are biased towards more severe injuries, since these patients are more likely to spend more time in the neuro-intensive care unit, hence being sampled more frequently. Also, sampling of NF-L was performed during different days, for all patients. Even if there were low intra-individual differences between samples, a more regular sampling protocol would have improved the study. However, considering the long half-life of NF-L and the low intra-individual sample difference, we do not think that the number of samples per patient constitutes a major limitation. Additionally, GCS was, surprisingly, not significantly correlated to outcome in our dataset, which leads to question the quality of the initial emergency room GCS. GCS at admission to the hospital has been shown to be influenced by sedative drugs, substance intoxication [67] and poor examination technique [68]. A motor GCS would have been preferable, as well as an objective post-resuscitation GCS. A statistical weakness of this study is that multiple testing was performed, and only the strongest correlations should therefore be discussed with confidence. It is also difficult to compare results from the different subpopulations (full, MRI, CSF-NF-L) as these differ somewhat in base-line variables. Since some patients had several CSF (and serum) samples, it was impossible to use a mixed-models approach in the correlations between serum and CSF concentrations of NSE and S100B (similar to NF-L in Fig 4A). As the release from CSF to serum might differ in individual patients, we believe that there are limitations to Fig 4A using the current method, even if the results in Fig 4A and 4B were similar for NF-L. All in all, this study must largely be seen as exploratory and hypothesis generating and we recommend further studies using fixed time points for sampling and imaging to better illustrate the correlation between NF-L and cerebral injury. Outcome was not assessed at the same time point for all patients, instead it was assessed over a time point of 6 months. Studies have shown that patients improve during this time period [69], and may even continue to improve after 12 months [70]. However, as it noted in the results, a majority of the patients were assessed at around 12 months after injury hence we believe that this is probably not a major limitation in our dataset.

## Conclusions

We found that NF-L levels in serum are significantly correlated to outcome even when adjusting for known strong predictors of TBI, including S100B. In line with its long half-life, logged NF-L levels remained relatively unchanged over time, albeit with an upward trend, exhibiting limited intra-patient differences. No correlation between DAI, or CT parameters, and NF-L levels could be detected. We hypothesize that NF-L might reflect a separate, more long term, pathophysiological process than current TBI biomarkers. We suggest that further studies are performed, with better quantification of axonal injury, and a prolonged sampling time, in order to better determine the correlation between NF-L and axonal pathology in TBI.

## Supporting Information

S1 File Raw data used in the study. Datasheets in a.zip-file containing all the patient data that were used to produce the findings in this study.
(ZIP)
